# Herbal Medicine Nanocrystals: A Potential Novel Therapeutic Strategy

**DOI:** 10.3390/molecules28176370

**Published:** 2023-08-31

**Authors:** Mengran Guo, Shugang Qin, Shiyan Wang, Min Sun, Huiling Yang, Xinchun Wang, Ping Fan, Zhaohui Jin

**Affiliations:** 1 Department of Pharmacy, West China Hospital, Sichuan University, Chengdu 610041, China; 2 Department of Critical Care Medicine, Frontiers Science Center for Disease-Related Molecular Network, State Key Laboratory of Biotherapy and Cancer Center, West China Hospital, Sichuan University, Chengdu 610041, China; 3 First Affiliated Hospital of the Medical College, Shihezi University, Shihezi 832008, China

**Keywords:** nanocrystals, herbal medicine, drug delivery, preparation, nucleation and polymorphism, applications

## Abstract

Herbal medicines have gained recognition among physicians and patients due to their lower adverse effects compared to modern medicines. They are extensively used to treat various diseases, including cancer, cardiovascular issues, chronic inflammation, microbial contamination, diabetes, obesity, and hepatic disorders, among others. Unfortunately, the clinical application of herbal medicines is limited by their low solubility and inadequate bioavailability. Utilizing herbal medicines in the form of nanocrystals (herbal medicine nanocrystals) has shown potential in enhancing solubility and bioavailability by reducing the particle size, increasing the specific surface area, and modifying the absorption mechanisms. Multiple studies have demonstrated that these nanocrystals significantly improve drug efficacy by reducing toxicity and increasing bioavailability. This review comprehensively examines therapeutic approaches based on herbal medicine nanocrystals. It covers the preparation principles, key factors influencing nucleation and polymorphism control, applications, and limitations. The review underscores the importance of optimizing delivery systems for successful herbal medicine nanocrystal therapeutics. Furthermore, it discusses the main challenges and opportunities in developing herbal medicine nanocrystals for the purpose of treating conditions such as cancer, inflammatory diseases, cardiovascular disorders, mental and nervous diseases, and antimicrobial infections. In conclusion, we have deliberated regarding the hurdles and forthcoming outlook in the realm of nanotoxicity, in vivo kinetics, herbal ingredients as stabilizers of nanocrystals, and the potential for surmounting drug resistance through the utilization of nanocrystalline formulations in herbal medicine. We anticipate that this review will offer innovative insights into the development of herbal medicine nanocrystals as a promising and novel therapeutic strategy.

## 1. Introduction

**Figure 1:**
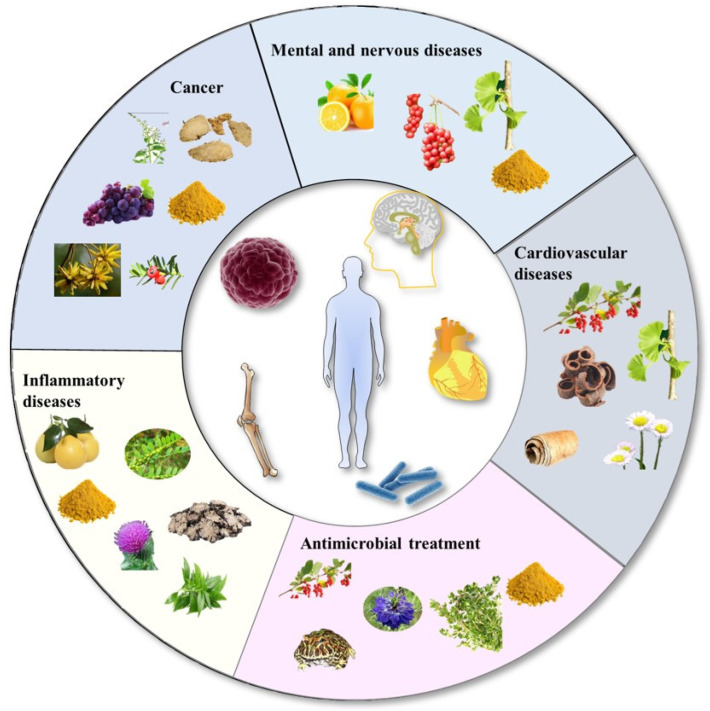
Application of herbal medicine nanocrystals in various diseases.

Herbal medicines have multiple active ingredients that exert therapeutic properties, such as antioxidant, anti-inflammatory, antitumor, antibacterial, and antiproliferative activities, and have been widely used to treat various diseases, including cancer, cardiovascular disease, chronic inflammatory disease, microbial contamination, diabetes, obesity, and liver disease (Figure 1) [1,2]. Therefore, herbal medicines provide active molecules for clinical therapeutic drugs [3], and almost one-third of the top-selling pharmaceuticals are derived from plants or other natural sources [3,4]. A large number of active ingredients of herbal medicines have been discovered and used in the clinic with the development of separation and extraction techniques. For example, the active ingredient paclitaxel is a natural secondary metabolite isolated and purified from the bark of *Taxus chinensis* (*Pilger*) *Rehd* and is widely used in the treatment of breast cancer, ovarian cancer, and non-small-cell lung cancer. Curcumin, another extensively researched active ingredient, is extracted from the root of *Curcuma longa* L. (*Zingiberaceae family*). It has favorable anti-inflammatory and antitumor effectiveness, and more than three hundred clinical trials have been conducted for its use in cancer [5] and atherosclerosis cardiovascular diseases [6]. The clinical application of herbal medicines is greatly limited by their low solubility and membrane permeability, short half-life, and inadequate bioavailability. Traditionally, herbal medicines have been prepared as tablets, capsules, and decoctions; unfortunately, these strategies did not improve the bioavailability of herbal medicines. Excitingly, nanotechnology has shown great achievements in enhancing the efficacy of herbal medicines and overcoming biological barriers by improving their bioavailability [7,8].

Nanocrystals have emerged as a popular nanodrug delivery system for hydrophobic drugs since the 20th century [9]. They are nanosized pure drug particles dispersed in an aqueous medium with particle sizes ranging from 10 to 1000 nm. Differently from other nanoformulations, nanocrystals do not require a carrier, which can minimize carrier-related toxicity. At the same time, the high drug load is beneficial for scaling up and increasing patient compliance. The most important advantages of nanocrystals for enhancing oral bioavailability include (1) increased saturation solubility and dissolution rate of poorly water-soluble drugs [10]; (2) enhanced adhesion to biological membranes, which prolongs the retention time in the gastrointestinal tract [11,12]; and (3) nanocrystals that can also be absorbed through intestinal epithelial cells [13] and the lymphatic pathway [14] with intact crystalline particles. However, during the preparation and storage processes, aggregation, Ostwald ripening, and sedimentation are prone to occurring due to the high surface energy and polymorphic transition, resulting in physical instability [15,16]. To solve these problems, key preparation processes and stabilizers such as surfactants and polymers should be considered to control nucleation and crystal growth and prevent collisions and aggregation between particles [15]. The preparation techniques for nanocrystals can be categorized into three main approaches: bottom-up methods, top-down methods, and combinations thereof. Following their preparation, nanocrystals can be employed either as liquid suspensions or advanced into tablet and capsule forms through solidification processes. Presently, a diverse range of nanocrystal-based drugs has entered the market. As per the available data, the US Food and Drug Administration (FDA) has received an excess of 80 applications encompassing Abbreviated New Drug Applications (ANDA), New Drug Applications (NDA), and Investigational New Drug (IND) submissions, all related to drug products incorporating nanocrystals [17]. This versatility allows for delivery via diverse routes of administration and application across a spectrum of diseases [17].

Given that a majority of the active compounds found in herbal medicines fall under the classification of the Biopharmaceutics Classification System (BCS) II or BCS IV, nano-crystals have emerged as a prominent tool for enhancing their oral bioavailability. This review succinctly encapsulates the existing benefits of herbal medicine nanocrystals, encompassing their preparation methodologies, pivotal factors governing nucleation and polymorphism, dissolution characteristics, and pharmacokinetic attributes, as well as recent advancements across diverse disease contexts. Ultimately, we delve into the challenges and future prospects entailing nanotoxicity, in vivo fate, and the potential for herbal medicine nanocrystals to overcome drug resistance.

## 2. Preparation Methods of Herbal Medicine Nanocrystals

**Figure 2:**
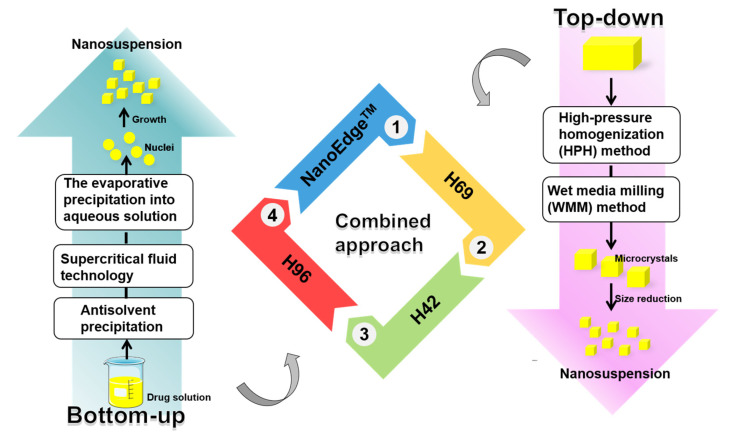
Nanocrystal preparation methods.

The preparation techniques for herbal medicine nanocrystals encompass bottom-up methods, top-down methods, and their amalgamation. We have synthesized these preparation methods, along with their respective merits and demerits, and they are shown in Figure 2 and Table 1.

### 2.1. Bottom-Up Methods

Bottom-up methods, often referred to as precipitation techniques, operate on the fundamental principle of precipitating drug nanocrystals from a solution that has reached a state of supersaturation. This process entails two distinct stages: nucleation followed by subsequent crystal growth [18]. The advantages of bottom-up methods are (1) simple to operate, (2) most cost-effective, and (3) more suitable for intravenous administration of anticancer compounds [18]. However, residual organic solvents may cause toxicity, and they are not suitable for drugs that are difficult to dissolve in organic solvents. In addition, the nucleation and crystal growth processes are difficult to control. The bottom-up methods primarily include antisolvent precipitation, supercritical fluid technology (SCF), and the evaporative precipitation into aqueous solution (EPAS) method.

#### 2.1.1. Antisolvent Precipitation

The antisolvent precipitation method entails the amalgamation of a drug solution, typically dissolved within an organic solvent compatible with water, with an aqueous antisolvent (often comprising stabilizers). This fusion engenders a state of supersaturation within the solution. Subsequently, the drug undergoes nucleation and precipitates, giving rise to nanoscale drug crystals. Stabilizers such as surfactants and polymers used to be added to limit the growth and aggregation of crystals. Curcumin (CUR) is a hydrophobic compound categorized within the BCS IV class, and it exhibits inherent constraints concerning solubility and bioavailability. Hyeonmin Lee et al. employed the antisolvent precipitation technique to fabricate curcumin nanocrystals, designated as TA-CUR-NSP. This process involved the utilization of stabilizers such as sodium dodecyl sulfate (SDS) and tannic acid-modified polyvinylpyrrolidone/vinyl acetate (PVP/VA). The curcumin (CUR) nanocrystals were successfully generated, achieving a particle size of 127.7 ± 1.3 nm. Notably, the mucoadhesion assay revealed that TA-CUR-NSP exhibited enhanced mucus affinity across all pH conditions. Furthermore, the drug’s plasma concentration for TA-CUR-NSP demonstrated a remarkable 7.2-fold increase in comparison to the pure drug formulation [19].

#### 2.1.2. Supercritical Fluid Technology

Supercritical fluid technology (SCF) involves the dissolution of drugs within a supercritical fluid. By pressurizing and atomizing the supercritical fluid through a nozzle with a small aperture, rapid vaporization occurs, leading to drug crystallization and the formation of drug nanocrystals. Carbon dioxide (CO_2_) stands as the predominant supercritical fluid choice due to its critical temperature of 31 °C and minimum critical pressure of 73.8 bar, facilitating its conversion into a supercritical state. This method is environmentally friendly, characterized by low costs, high safety, and facile removal of the supercritical fluid [20].

#### 2.1.3. Evaporative Precipitation into Aqueous Solution Method

The evaporative precipitation into aqueous solution (EPAS) method was first developed by Sarkari et al. [21] and is now widely used to fabricate nanocrystals of poorly water-soluble drugs [22,23]. It employs rapid phase separation to initiate nucleation and foster the growth of nanocrystals for poorly soluble drugs. In essence, the drug is dissolved in an organic solvent with a low boiling temperature, such as dichloromethane. This solution is then sprayed through a fine nozzle into a heated aqueous solution. Rapid evaporation of the organic solvent occurs, leading to the drug’s precipitation alongside polymeric surfactants acting as stabilizers [21]. Artemisinin (ART) is a highly potent antimalarial medication effective against multi-drug-resistant Plasmodium falciparum malaria strains. Unfortunately, its clinical efficacy is hindered by its limited solubility. In a study by Mitali Kakran et al., an artemisinin nanocrystal was engineered through the evaporative precipitation into aqueous solution (EPAS) technique, employing PEG as a stabilizing agent. The investigation revealed that the crystalline nature of artemisinin in the nanocrystals diminished proportionally with the escalating polymer concentrations. Notably, the drug’s solubility and dissolution rate exhibited significant enhancements in comparison to the original drug powder [24].

### 2.2. Top-Down Methods

Top-down methods involve the fragmentation of large crystals into nanoscale particles through the application of mechanical force. The notable benefits of employing these techniques include the elimination of the requirement for organic solvents and the simplicity of scaling up production processes [25]. These primarily encompass the wet media milling (WMM) method and the high-pressure homogenization (HPH) method.

#### 2.2.1. Wet Media Milling

In the early 1990s, Merisko Liversidge et al. introduced the wet media milling method (i.e., Nanocrystal^TM^, Alkermes PLC, Waltham, MA, USA) to reduce the insoluble drug particle size [26]. This technology has witnessed rapid development owing to its uncomplicated preparation procedure and minimal excipient requirement. Typically, a liquid blend of the crude drug and stabilizers, in specified proportions, is introduced into a chamber filled with grinding media. These grinding media materials commonly encompass zirconia, glass, agate, or beads coated with polystyrene resin. Through high-speed rotation, interactions arise among drug particles, grinding media, and the container walls, engendering persistent and robust pressure and shear forces. These forces supply the necessary energy for the micronization of the drug particles. This method presents several advantages, including its straightforward preparation process and the attainment of a narrow particle size distribution for the resultant nanocrystals. These features render it particularly suitable for drugs that exhibit insolubility in both water and organic solvents. Moreover, its noteworthy benefits extend to various drug delivery pathways, including oral and intravenous administration. Among these, oral administration stands out, attracting the most attention due to its pronounced advantages. Denisa Lizoňová and her team successfully fabricated curcumin nanocrystals through the utilization of a wet media milling technique, yielding a particle size range of 40–90 nm. They opted for a synergistic blend of phospholipids and polyethylene glycol as stabilizers, which proved to be the most effective combination, bestowing a steric stabilization effect. This formulation resulted in colloidal stability across diverse media and exhibited remarkable anticancer potential in comparison to widely employed commercial cytostatic agents [27].

#### 2.2.2. High-Pressure Homogenization

High-pressure homogenization (HPH) stands as the second-generation nanocrystal preparation technology, having been pioneered by Müller et al. in the 1990s, succeeding techniques such as Nanopure^TM^ (SkyePharma PLC, Saint-Quentin-Fallavier, France). This method can be categorized into microfluidization technology (e.g., Insoluble Drug Delivery Particles, IDD-PTM) and piston-gap homogenization technology (DissoCubes^TM^, Abbott GmbH &Co., KG, Wiesbaden, Germany).

In microfluidization technology, a drug suspension is swiftly propelled through the homogenization chamber using jet air, subjecting it to repeated directional shifts within the pipeline. This intricate process invokes effects like cavitation, impact, and shear, effectively reducing the sizes of the drug particles.

In piston-gap homogenization technology, an insoluble drug previously subjected to micronization is transformed into a crude suspension. This suspension is then propelled through a high-pressure homogenizer pump, passing through the homogenization valve gap at high speed. This process culminates in the formation of final nanocrystals. The sizes of the resultant particles can be precisely controlled by optimizing the high-pressure homogenization cycle, which often requires several iterations.

High-pressure homogenization finds extensive application in herbal medicines. For instance, Lou et al. successfully developed oridonin nanocrystals (ORI-N) through high-pressure homogenization. ORI-N displayed a particle size of 897.2 ± 14.2 nm and a zeta potential of −21.8 ± 0.8 mV. Notably, ORI-N exhibited potent inhibition against SMMC-7721 cell proliferation, leading to a higher apoptotic rate compared to the bulk ORI solution. Furthermore, ORI-N demonstrated superior antitumor efficacy, underscoring nanocrystal formation as a promising strategy for enhancing the therapeutic potential of oridonin in tumor treatment [28].

### 2.3. Combinative Technology

Attaining nanocrystals with exceedingly reduced particle sizes via a sole technology presents challenges. Consequently, combined techniques have been devised. These integrated approaches amalgamate the bottom-up and top-down methodologies. Typically, the precipitation method is applied to create initial coarse suspensions, followed by the milling method for precise particle size regulation and uniformity in the ultimate nanocrystals.

Prominent examples of such combinative technologies include the NanoEdge™, H69, H42, and H96 technologies. These techniques capitalize on the synergy between the strengths of both bottom-up and top-down approaches, ultimately enhancing the control and optimization of nanocrystal properties.

#### 2.3.1. NanoEdge™

NanoEdge™ (Baxter, IL, USA), also referred to as the precipitation homogenization method, stands as one of the pioneering combined technologies for the preparation of nanocrystals. In this process, the drug undergoes both nucleation and crystal growth. The majority of crystals formed at this stage tend to be imperfect. Some may adopt an amorphous or partially crystalline state, a quality advantageous for subsequent high-pressure homogenization procedures.

A notable example is the work of Yang et al., who successfully developed a 10-hydroxycamptothecin (10-HCPT) nanocrystal. They employed a modified acid–base microprecipitation approach coupled with a high-pressure homogenization technique, resulting in nanocrystals with particle sizes of approximately 130 nm. This method showcases the potential of combining distinct approaches to tailor nanocrystal properties [29]. This nanocrystal had higher cellular uptake and antiproliferative activity than the 10-HCPT injections. It also enhanced the antitumor efficacy.

#### 2.3.2. H69 Technology

H69 technology integrates the cavitation precipitation and high-pressure homogenization methods. A key distinction between H69 technology and NanoEdge™ lies in the timing of events. In the H69 process, precipitation and high-pressure homogenization take place almost concurrently. As drug crystals emerge during the precipitation stage, they are immediately exposed to cavitation, enduring the impact force and shear force of the homogenizer. This process serves to finely regulate crystal growth and enhance the uniformity of the resulting nanocrystals.

#### 2.3.3. H42 Technology

H42 technology merges the principles of spray drying and high-pressure homogenization. In the H42 process, the hydrophobic drug is initially dispersed within stabilizers via a spray drying procedure. Subsequently, this drug–stabilizer mixture is dispersed into an aqueous solution, leading to the formation of a coarse suspension. This suspension then undergoes high-pressure homogenization, which regulates the particle size. While H42 technology finds extensive application in commercial production, it is worth noting that its yield tends to be low, and it may not be suitable for drugs sensitive to heat.

#### 2.3.4. H96 Technology

H96 technology encompasses the combination of freeze drying and high-pressure homogenization. Analogous to H42 technology, the initial step involves the pretreatment of the crude drug via freeze drying. This process imparts a loose and porous structure to the drug crystals, rendering them more susceptible to further fragmentation. A noteworthy feature of this method is the ability to bypass the requirement for organic solvents while still achieving optimal nanocrystal formation under relatively mild conditions. However, it is important to acknowledge that the freeze-drying procedure does demand a considerable amount of time.

### 2.4. Key Factors to Control Nucleation and Polymorphism

#### 2.4.1. Polymorphism Transformation in the Top-Down Method

Polymorphism transformation significantly affects the dissolution characteristics of nanocrystals. Amorphous nanocrystals inherently exhibit favorable dissolution rates without necessitating the overcoming of lattice energy. In the context of the “top-down” approach, milling processes are primarily propelled by factors such as cavitation forces, shear forces, and inter-particle collisions within the chamber. The mechanical energy’s pressure can disturb crystal lattices, potentially leading to the transformation of the drug crystal into an amorphous state or a metastable form [30]. The process of polymorphic transformation during mechanical milling typically involves two steps: (1) the amorphization of the impacted fraction and (2) rapid recrystallization guided by surrounding crystallites, favoring the γ or α forms [31]. Notably, Mazel et al. observed that anhydrous caffeine underwent a transformation from form I to a metastable state following grinding, eventually transitioning into form II over a period of days [32].

Milling temperature, along with the heat generated during milling, can further heighten the probability of crystal transformation. Amorphization becomes likely when the milling temperature substantially deviates from the glass transition temperature (Tg). Near Tg, recrystallization may manifest on the surface due to heightened molecular mobility, surpassing that within the bulk. Additionally, temperatures exceeding Tg tend to induce the emergence of new crystal forms [30,33].

While particle size reduction and amorphization are anticipated to enhance nanocrystal dissolution behavior, they can also introduce instability concerns. The creation of crystal surface defects and amorphization during milling leads to elevated specific surface energy in nanocrystals. Stabilizers can be introduced to embed within defect lattices and amorphous drug molecules, mitigating excessive surface energy and preserving particle stability. Consequently, optimizing preparation parameters such as milling speed, pressure, time, cycles, temperature, and stabilizer incorporation becomes pivotal to achieving optimal outcomes.

#### 2.4.2. Nucleation and Polymorphism Transformation in the Bottom-Up Method

The “bottom-up” method engenders nanocrystals at the molecular level of the drug within a solution. This process involves the sequential occurrences of nucleation and crystal growth. The pivotal factors governing nucleation and crystal growth encompass molecular diffusion, supersaturation, surface tension, temperature [34], solvent [35] and stabilizers [36]. When an insoluble drug is mixed with an antisolvent solvent, a high metastable state situated at a supersaturation level is achieved [37,38]. This condition leads to swift nucleation bursts characterized by gradual molecular diffusion and pronounced interfacial tension. The abundance of nuclei produces results in a heightened surface area, accompanied by elevated free energy. To minimize the total surface energy, particles tend to amalgamate through flocculation aggregation and crystal growth. However, this process introduces impurities, demands high energy consumption, and carries the potential for polymorphic transformation [39]. The meticulous optimization of organic solvents and stabilizers is crucial to controlling nucleation and crystal growth. Stabilizers, including polymers and surfactants, have the capacity to attach to the surfaces of nuclei, restraining crystal growth. These stabilizers additionally enhance stability through spatial hindrance and electrostatic repulsion. The specific type and concentration of stabilizers dictate the crystallinity and melting point. Lennart et al. demonstrated that even a minute concentration of PVP could significantly diminish the crystal growth rate and modify the crystal morphology [40].

Upon simultaneous implementation of the “top-down” and “bottom-up” methods, distinct and controlled nuclei are generated, preventing them from maturing into larger nuclei.

## 3. Characterization of Nanocrystals

### 3.1. Particle Size

The Noyes–Whitney equation (Equation (1)) establishes a direct relationship between the dissolution rate of a drug and the surface area of its particles. As particle size decreases, the dissolution rate increases. Thus, particle size stands out as a pivotal attribute for assessing nanocrystals. In a study by Wang et al., the impact of particle size on the in vitro and in vivo behavior of astilbin nanocrystals (AT-NSs) was investigated. They formulated three AT-NSs with particle sizes of 215.17 ± 4.83 nm, 496.51 ± 12.94 nm, and 803.34 ± 9.06 nm. The formulation featuring a particle size of 215.17 nm exhibited a notably enhanced dissolution rate. However, the formulation with a particle size of 496.51 nm displayed the highest bioavailability. This disparity could be attributed to larger AT-NSs being sequestered by the reticuloendothelial system, accumulating in the lung and liver [41].

In another investigation, two silybin nanocrystals were prepared, with particle sizes of 637 ± 9.4 nm (SN-A) and 132 ± 4.8 nm (SN-B). Intriguingly, SN-A, when intravenously administered, exhibited a preference for targeting the liver and spleen [42]. SN-B, on the other hand, demonstrated superior induction of PC-3 cell apoptosis compared to SN-A [43]. These findings underscore the significant influence of particle size not only on the dissolution rate, but also on the biodistribution of nanocrystals.

*d*C*/d*t *=* AD/*d*(C_s_ − C_x_) (1)

where *d*C/*d*t is the dissolution rate; A is the surface area of the drug particle; D is the diffusion coefficient; *d* is the diffusion layer thickness; C_s_ is the saturation solubility of the drug; and C_x_ is the mass concentration of the drug in the solvent.

### 3.2. Solid State

During the preparation of nanocrystals, the drug crystals can exist in either a crystalline or amorphous state [44]. In the case of amorphous drug nanocrystals, the need to overcome lattice energy upon drug molecule dissolution is eliminated, resulting in a heightened dissolution rate. However, the amorphous state represents a high-energy condition, potentially leading to physical and chemical stability issues [45]. Gye Hwa Shin et al. successfully created an amorphous curcumin-TPGS nanocrystal through ultrasonic homogenization to enhance curcumin’s solubility. This formulation showcased a remarkable 433-fold improvement in water solubility compared to crude curcumin [46]. In another study, Liu et al. examined the solid-state characteristics of resveratrol nanocrystals prepared using different methods, including spray drying, rotary evaporation, and quench cooling, all coupled with high-pressure homogenization. Their findings highlighted that spray-drying pretreatment marginally reduced the crystallinity and altered the morphology of resveratrol nanocrystals, ultimately leading to the smallest particle size [47].

### 3.3. Morphology

The morphology of nanocrystals varies based on the preparation methods or stabilizers employed. Stabilizers have been shown to influence crystal growth rates in distinct directions [48], yielding diverse morphologies, such as needle-like, spherical, flaky, cubic, and rod-like crystals. This phenomenon significantly impacts the dissolution and bioavailability of nanocrystals [11].

## 4. Application of Nanocrystals in Herbal Medicines

**Figure 3:**
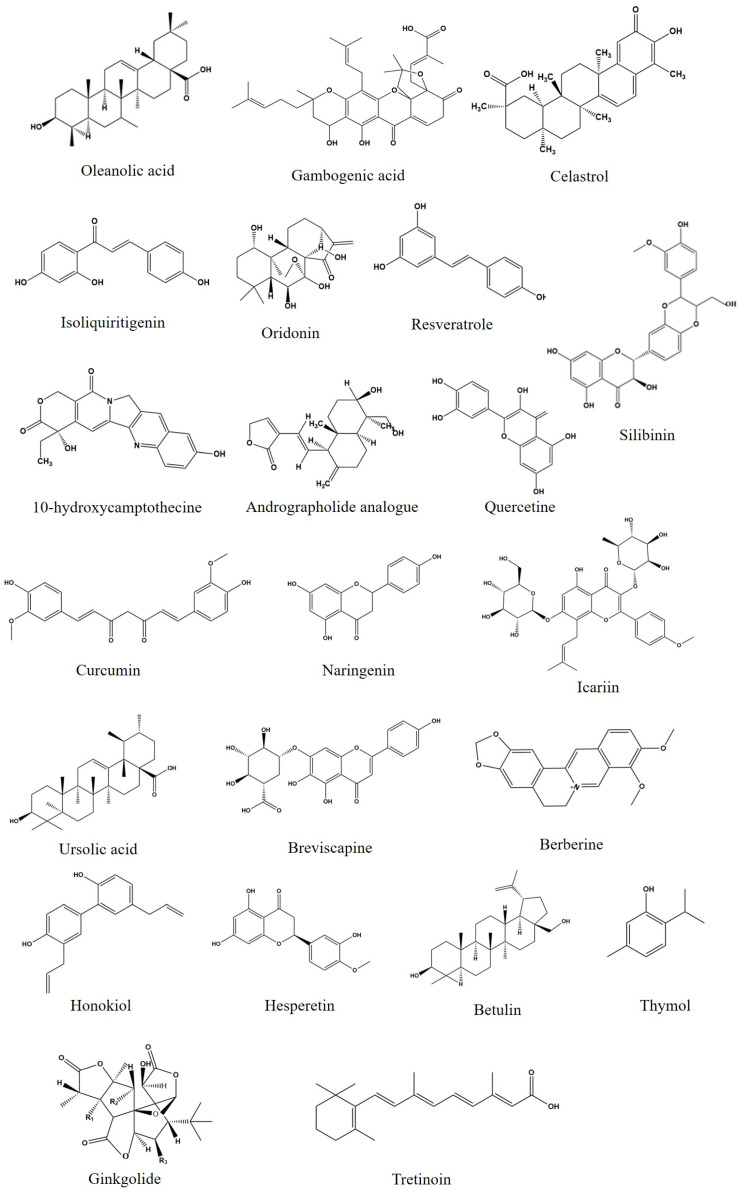
Chemical structures of some of the main herbal medicine active ingredients.

This section offers an overview of the application of herbal medicine nanocrystals for diverse diseases, including cancer, inflammatory diseases, cardiovascular diseases, mental and nervous diseases, and antimicrobial diseases. Figure 3 illustrates the chemical structures of several active ingredients found in herbal medicines.

### 4.1. Cancer Therapy

In recent decades, herbal medicines have garnered substantial attention due to their potent biological activities, including antiproliferation, antioxidant effects, apoptosis induction, and antitumor properties, rendering them promising agents for cancer therapy. Nonetheless, many active constituents in herbal medicines fall under the BCS II or BCS IV categories, characterized by poor water solubility and membrane permeability which hinder therapeutic efficacy. Nanocrystal technology emerges as a promising strategy to address these challenges. The application of nanocrystals in herbal medicines for cancer therapy is outlined in Table 2.

Annonaceous acetogenins (ACGs) are prominent antitumor agents extracted from seeds of the *Annonaceae* species [49]. They exhibit significant antitumor activity against various cancer cell lines, including A549, MCF-7, L1210, SMMC7721, HeLa, MKN-45, and HepG2 [49,50,51,52,53]. Despite their efficacy, ACGs may cause acute toxicity upon oral administration, for example, liver and kidney damage [54,55]. To enhance ACGs’ antitumor effectiveness while mitigating toxicity, Hong et al. produced an ACG nanocrystal (ACG-NSps) using an antisolvent sonoprecipitation technique. Employing mPEG2000-PCL2000 as a stabilizer, the resulting ACG-NSps exhibited particle sizes of 123.2 ± 3.54 nm and a zeta potential of −20.17 mV. This formulation demonstrated enhanced cytotoxicity against 4T1, MCF-7, and HeLa cells, superior to the free drug. Notably, ACG-NSps exhibited efficient tumor accumulation and achieved enhanced therapeutic efficacy with a mere 1/10th of the dose compared to the drug oil solution [50].

Gambogenic acid, a key component of *Garcinia hanburyi*, displays a broad antineoplastic spectrum across breast cancer, lung cancer, and multiple myeloma [56,57,58,59]. It holds the potential to reverse P-gp-mediated multidrug resistance and influence apoptosis through ROS-dependent activation of IRE1α/JNK [60]. To enhance the bioavailability and antitumor efficiency of gambogenic acid, Yuan et al. developed gambogenic acid nanocrystals (GNA-NSs) with PVP K30 and PEG_2000_ as stabilizers, resulting in particles sized at 183.7 nm with a zeta potential of −22.8 mV. The AUC_0–∞_ and t_1/2_ of GNA-NSs were increased by 2.63-fold and 1.77-fold compared with those of the reference formulation, along with elevated cytotoxicity in HepG2 cells [61].

10-Hydroxycamptothecin is an alkaloid extracted from the seeds or roots of *Camptotheca acuminata Decne.* It can selectively inhibit topoisomerase I, interfering with DNA replication [62], and is widely exploited in clinical practice [63]. Although it has poor solubility in water, it can easily dissolve in a basic solution and precipitate after adding an acidic solution. Utilizing a modified acid–base microprecipitation technique coupled with high-pressure homogenization, Yang et al. created a 10-hydroxycamptothecin nanocrystal free of organic solvents. With a spherical particle size of around 130 nm, this nanocrystal demonstrated improved tumor accumulation and anticancer efficacy compared to 10-hydroxycamptothecin injection [29]. Zhou et al. compared the anticancer activities of hydroxycamptothecin nanocrystals with different shapes (nanorods and nanoparticles). They proved that nanorods showed higher and longer-term toxicity than nanoparticles [64], indicating that particle shape is also an important characteristic for nanocrystals.

Resveratrol, an extract from grapes, has gained attention as a potential antitumor drug and chemopreventive agent [65] due to its cancer initiation, promotion, and progression effects [65]. It was reported that resveratrol could inhibit cell proliferation by blocking PI3k/Akt/mTOR [66]. It could also downregulate CD8^+^CD122^+^ Tregs to inhibit hepatocellular carcinoma. Wang et al. crafted a folate-modified resveratrol nanocrystal, enhancing cytotoxicity against A549 cells and amplifying antitumor efficacy [67]. Additionally, a recent study revealed that resveratrol nanocrystals, when compared to their non-nanocrystalline forms, demonstrated significant reductions in tumor cell proliferation, fewer blood vessels in the peritoneum, and diminished systemic toxicity (such as hepatocellular necrosis and apoptosis, hepatic fibrosis, and minor fatty changes) in Ehrlich ascites tumor-bearing mice [68].

**Table 2:** Summary of nanocrystal formulations used in herbal medicines for cancer therapy.

Extract/Compounds	Stabilizers	Preparation Methods	Particle Size	Bioavailability	Advantages	Applications	Ref.
Annonaceous acetogenins	mPEG_2000_–PCL_2000_	Antisolvent sonoprecipitation	123.2 ± 3.54 nm	/	Nanocrystal achieved much better therapeutic efficacy than the traditional dosage form (oil solution)	Antitumor	[50]
Oridonin	Lecithin, HPMC, and PVP	High pressure homogenization	912.5 ± 17.6 nm	/	Significantly inhibited the proliferation of PC-3 cells; enhanced growth suppression; and induced apoptosis of PC-3 cells, higher antitumor efficacy, lower toxicity	Prostatic carcinoma	[28,69]
Gambogenic acid	PVPK30 and PEG_2000_	Antisolvent precipitation	183.7 nm	AUC and t_1/2_ of GNA-NS were increased 2.63- and 1.77-fold than that of the reference formulation	Exhibited superior cytotoxicity compared with GNA solution toward HepG2 cells	Antitumor	[61,70]
Isoliquiritigenin	HPC SSL and PVP K30	Wet media milling	238.1 ± 4.9 nm and 354.1 ± 9.1 nm	/	Improved the solubility; enhanced the cytotoxicity	Antitumor	[71]
Oleanolic acid	Sucrose monolaurate and sucrose monopalmitate	O/W emulsion and organic solvent evaporation methods	~100 nm	Oral bioavailability of the oleanolic acid nanocrystal was 6–7-times higher than that of the oleanolic acid coarse suspension	NS group had significantly higher bioavailability (6- to 7-fold) than the suspension group	Anticancer	[72]
Silibinin	Lecithin and poloxamer 188	High pressure homogenization	641.8 ± 14.7 nm 127 ± 1.9 nm	/	Showed better apoptosis effect on PC-3 cells	Prostatic carcinoma	[43]
Celastrol	P 188	Antisolvent precipitation	147.9 nm	/	Displayed a significantly enhanced tumor inhibition rate and therapeutic efficacy in comparison with that of the CSL suspension.	Breast cancer	[73]
10-hydroxycamptot-hecin	P 188	A modified acid–base microprecipitation combined with a high-pressure homogenization technique	~130 nm	The AUC_0–24_ value was 2.81-fold, as high as the injections group. Meanwhile, the mean residence time of the 10-HCPT nanocrystal was significantly higher than that of the 10-HCPT injections group.	10-HCPT nanocrystals exhibited much higher drug levels in the plasma and tissues of the test mice than the marketed 10-HCPT injections, and significantly improved the antitumor therapeutic effect.	Antitumor	[29,74]
Quercetin	Polysorbate 80	Nanoprecipitation and high-pressure homogenization	393.5 nm	Exhibited a significant reduction in clearance rate and increase in AUC compared with the control suspension.	The solubility of QT in nanocrystals was approximately 70-fold that of crude QT, and the dissolution of QT from QT-NS was increased as compared to that of the original QT powder.	Antitumor	[75]
Andrographolide analogue (3A.1)	Chitosan derivatives	Antisolvent precipitation	220–270 nm	/	Increased solubility and pharmacological effectiveness with the induction of apoptosis; had the strongest anticancer effect compared to the drug solution	Colorectal cancer	[76]
Resveratrol	TPGS and DSPE-PEG-FA	Antisolvent precipitation	100–200 nm	/	Higher antitumor efficacy due to reduced tumor volume and weight	Antitumor	[67]
Curcumin	mPEG2000-DSPE and soybean lecithin	Antisolvent sonoprecipitation	186.33 ± 2.73 nm	After i.v. administration, the AUC_0–24_ of CUR-NSps was 4.50 times that of the CUR injections; t_1/2_ of CUR-NSps was approximately 35.95 times that of CUR injections. The mean residence time of CUR-NSps was 18.90-fold longer than that of CUR solution.	CUR-NSps exhibited a significantly greater AUC_0–24_ and prolonged MRT compared to CUR injections after i.v. administration. Higher biodistribution in the liver, kidney, brain, and tumor for CUR-NSps compared to CUR injections.	Antitumor	[77]
Curcumin	TPGS	Trituration followed by ultraturrax homogenization and high-pressure homogenization	210.2 nm	AUC_0–∞_ of CUR-NS was approximately 3.8-fold greater than CUR solution; the mean residence time was 11.2-fold longer.	CUR-NS showed greater AUC_0–∞_ and prolonged MRT compared to CUR solution in rabbits after i.v. administration.	Antitumor	[78]

### 4.2. Inflammatory Diseases

Inflammation serves as a key pathological basis or complication in various disorders, including osteoarthritis, pulmonary ailments, and hepatic conditions. Herbal medicines often possess anti-inflammatory properties, making them valuable candidates for intervention. For instance, naringenin demonstrates the capacity to regulate intracellular cytokine degradation, aiding in the amelioration of acute inflammation [79]. Icariin, on the other hand, inhibits the HO-1/Nrf2 and NF-κB signaling pathways to mitigate carrageenan-induced acute inflammation [80]. Additionally, silymarin acts as a hepatoprotective agent, and is capable of mitigating liver damage induced by diet or drugs [81,82]. The summary of herbal medicine nanocrystals for inflammatory diseases was listed in Table 3.

Gera et al. reported on naringenin nanocrystals (NRG-NS) produced through antisolvent sonoprecipitation, resulting in a particle size of 117 ± 5 nm. These nanocrystals exhibited heightened ALP levels compared to NRG and demonstrated superior structural repair of the cortical and trabecular bone architecture [83]. In a separate study, NRG nanocrystals were developed via the wet media milling technique with TPGS to address postinfectious cough [84]. The NRG nanocrystal showcased enhanced antitussive effects, yielding a 3-fold and 1.6-fold reduction in cough frequency compared to the blank model and crude NRG, respectively.

Icariin, a BCS IV flavonoid glycoside, faces solubility and membrane permeability limitations that hinder oral absorption. Icaritin, the bioactive aglycone form and a major intestinal metabolite of icariin, falls under BCS II compounds, boasting better potency than icariin in terms of osteoblast differentiation and proliferation. Li et al. crafted icaritin nanocrystals (ICTN) using the antisolvent precipitation method, yielding rod-shaped particles with sizes of 216.6 ± 12.4 nm and an approximate 50% decrease in crystallinity. Bioavailability studies unveiled significantly elevated C_max_ (4.7-fold higher) and AUC_0–12_ (2.0-fold higher), along with decreased T_max_, compared to unformulated ICT. The study on anti-osteoporosis activity further demonstrated that ICTN improved osteoblast proliferation and differentiation, whereas ICT did not elicit such stimulation [85].

Li et al. engineered tetramethylpyrazine dihydroxynaphthalenate nanocrystals (TMP-NS) for intra-articular injection to target osteoarthritis [86]. TMP-NS exhibited retention lasting five times longer in the articular cavity, elevated TMP concentrations in the joints, and more pronounced anti-osteoarthritic efficacy compared to the TMP solution.

**Table 3:** Summary of nanocrystal formulations used in herbal medicines for inflammatory diseases.

Extract/Compounds	Stabilizers	Preparation Methods	Particle Size	Bioavailability	Advantages	Applications	Ref.
Curcumin	P188	Wet media milling	200–240 nm	/	/	Bronchial Asthma	[87]
Naringenin	PVP K90	Antisolvent sonoprecipitation	117 ± 5 nm	/	NRG-NS showed a decrease in the levels of acid phosphatase and inorganic phosphorus compared to plain NRG	Anti-osteoporotic	[83]
Naringenin	TPGS	Wet media milling	~182.2 nm	/	Nanocrystal showed an excellent antitussive effect. The cough frequency decreased by threefold compared with the blank model, with an inhibition rate of 66.7%. Showed a good cough-relieving effect, decreasing IL-6 and MDA, increasing SOD, and reducing lung damage	Post-infectious cough	[84]
lipophilic aglycone icaritin	HPMC E3	Antisolvent precipitation	216.6 ± 12.4 nm	2.0-fold in AUC_0–12_ and 4.7-fold in C_max_	ICTN exhibited a faster dissolution rate, significantly faster absorption. Enhanced proliferation and differentiation activities.	Treats impotence and prevent osteoporosis	[85]
Phyllanthus amarus extract	1.5% PVA	Nanoprecipitation method	243 ± 9.7 nm	/	The levels of the serum enzymes and bile salt were significantly lower.	Hepatic disorders	[88]
Silymarin	PVA	Antisolvent sonoprecipitation	277.3 ± 10.4 nm	/	Exhibited faster dissolution rate, higher drug content, and pronounced enhancement of saturation solubility.	Liver disorders, for instance, acute and chronic viral hepatitis, cirrhosis, toxic hepatitis and fatty liver	[89]
Tetramethylpyrazine	0.2% PVP K30 and 0.8% HPMC	Wet media milling	588 nm	The t_1/2_ of TMP-NS was approximately three times longer than in the TMP group. The average retention time of TMP-NS was approximately five times longer than TMP.	After intra-articular injection in rats, NS had a longer retention time in the articular cavity, higher TMP concentrations in the joints, and greater anti-osteoarthritic efficacy than the TMP solution.	Osteoarthritis	[86]
Andrographolide	TPGS	Wet media milling	244.6 ± 3.0 nm	The AUC_0–t_ and C_max_ of the freeze-dried ADG-NS were threefold and twofold higher than the ADG coarse powder. The AUC_0–t_ of the freeze-dried ADG-NS was 54.3% higher than the freeze-dried ADG-NS without TPGS. The AUC_0–t_ of the freeze-dried ADG-NS (with or without TPGS) was 1.38- or 2.14-fold higher than that of the ADG dripping pills.	ADG-NS showed higher permeability and plasma exposure. ADG-NS were more effective in reducing the rate of paw swelling and producing a greater increase in the serum levels of nitric oxide (NO), interleukin-1 (IL-1), and tumor necrosis factor-α (TNF-α), as well as an increase in superoxide dismutase activity	Anti-inflammatory	[90]

### 4.3. Cardiovascular Diseases

Herbal medicines have a long history of utilization in addressing cardiovascular diseases, offering various benefits such as blood pressure reduction, lipid lowering, glucose regulation, myocardial protection, and anti-platelet aggregation effects [91,92,93]. To enhance the bioavailability and therapeutic efficacy of insoluble active ingredients, nanocrystals have been harnessed. For instance, honokiol is derived from the stem bark of Magnolia officinalis Rehd. Et Wils, and exhibits platelet aggregation inhibitory effects by acting as a specific collagen receptor glycoprotein VI antagonist on human platelets [94]. Additionally, it plays a role in regulating mitochondrial substrate utilization and cellular fatty acid metabolism in diabetic mouse hearts [95,96]. To improve its oral bioavailability and distribution within the cardiovascular and cerebrovascular systems, Han et al. developed honokiol nanocrystals (HK-NSps) with particle sizes of 116.2 ± 1.77 nm [97]. Using PVP and BSA as stabilizers, they achieved an approximate 3.94-fold increase in C_max_ and a 2.2-fold increase in AUC_0–t_ of HK-NSps compared to Honokiol coarse suspension after oral administration at 20 mg/kg body weight. This enhancement stemmed from improved dissolution, adhesion between nanoparticles and intestinal villi, and specific absorption pathways like lymphatic transport. Biodistribution investigations indicated heightened C_max_ and AUC_0–t_ in the liver and kidney following oral administration of HK-NSps. Intravenous administration of HK-NSps further elevated their distribution in the heart and brain, signifying the promising potential of HK-NSps in the treatment of cardio-cerebro-vascular ailments. Various herbal medicine nanocrystals for cardiovascular diseases are summarized in Table 4.

### 4.4. Mental and Nervous Diseases

Addressing brain disorders, including mental and nervous diseases, poses significant challenges. Neurodegenerative diseases such as Alzheimer’s disease (AD) and Parkinson’s disease (PD) involve progressive neurodegeneration in the brain. Existing drugs for these conditions often exhibit limited efficacy and substantial side effects, which are less than ideal for clinical use. Herbal medicines have promise for the treatment of neurodegenerative diseases by regulating mitochondrial dysfunction-induced cell apoptosis [103] and influencing critical signaling pathways like PI3K, NF-κB, and AMPK, which are associated with oxidative and inflammatory stress in neurons [104]. Nevertheless, due to the poor water solubility of active components in herbal medicines, their transport across the blood–brain barrier (BBB) to achieve effective concentrations is challenging. Nanocrystals present a strategy by which to increase drug accumulation in the brain by enhancing blood drug concentrations. The application of nanocrystals in mental and nervous system diseases is presented in Table 5.

Ginkgolide B (GB) is a potent anti-Parkinsonism compound known for its ability to reduce reactive oxygen species [105] and inhibit nerve cell apoptosis [106]. Given GB’s low solubility (2.5 × 10^−4^ mol/L), its in vivo drug concentration is often insufficient. To address this, Liu et al. developed ginkgolide B nanocrystals (GB-NCs) with particle sizes of 83.48 ± 1.77 nm. In rat models, GB-NCs exhibited elevated drug plasma levels and enhanced neuronal drug distribution (see Table 5), leading to shorter T_max_ and t_1/2_ compared to coarse drugs. Due to its transport across the gastrointestinal tract and BBB, GB-NCs demonstrated a much longer MRT in the brain compared to plasma. This favorable trait of GB-NCs improved behavioral deficits and reduced dopaminergic deficiency, highlighting their potential as an efficient means for facilitating brain delivery of GB [107].

Alzheimer’s disease, a prevalent neurodegenerative condition among the elderly, is characterized by a high incidence rate. Stahr et al. developed hesperetin nanocrystals with varying particle sizes and examined the impact of particle size on Alzheimer’s disease treatment. Decreasing the particle size, particularly below 200 nm, led to an increased dissolution rate [108].

**Table 5:** Summary of nanocrystal formulations used in herbal medicines for mental and nervous system diseases.

Extract/Compounds	Stabilizers	Preparation Methods	Particle Size	Bioavailability	Advantages	Applications	Ref.
Quercetin	/	Evaporative precipitation	120 nm	/	Increased antioxidant enzyme activities	Parkinson’s disease	[109,110]
Schisantherin A	0.1% HPMC E3	Antisolvent precipitation	∼160 nm	In plasma, SA-NC increased 7.88-fold in C_max_ and 6.37-fold in AUC_0–t_. In the brain, SA-NC increased by 5.47-fold in C_max_ and 6.32-fold in AUC_0–t_.	A fast dissolution rate in vitro, Higher concentration in plasma and brain; higher efficiency in reversing MPTP-induced dopaminergic neuronal loss and locomotion deficiency in zebrafish, as well as the MPP+-induced damage of neuronal cell culture model; stronger neuroprotective effect	Parkinson’s disease	[111]
Ginkgolide B	0.05% HPMC E5	Antisolvent precipitation	83.48 ± 1.77 nm	In plasma, GB-NCs increased by 13-fold in C_max_ and by 5-fold in AUC_0–t_. GBNCs had a shorter T_max_ and t_1/2_. In the brain, GB-NCs increased by threefold in C_max_, and by 2.5-fold in AUC_0–t_. GBNC increased T_max_ and delayed t_1/2_. The MRT_0–t_ of the GB-NCs in the brain were 2.11-fold longer than those of the plasma.	GB-NCs have high rates of dissolution, enhanced cellular uptake, and permeability; higher concentrations in the plasma and brain; remain for longer times in the brain; and possess higher efficiency in protecting neurons against cytotoxicity induced by MPP+, The GB-NCs can protect neurons against cytotoxicity induced by MPP+, improve behavior, reduce dopamine deficiency, and elevate dopamine metabolite levels.	Parkinson’s disease	[107]
Hesperetin	Plantacare 2000	High-pressure homogenization and wet media milling	Between 200 and 800 nm	/	Increased dissolution rate and kinetic solubility, higher antioxidant capacity	Alzheimer’s disease	[108,112]
Curcumin didecanoate	5% F68	Wet media milling	517 ± 9 nm	3.73-fold in C_max_, 4.7-fold in AUC_0–t_.	Higher concentration in brain	Antidepressant	[113]

### 4.5. Antimicrobial Treatment

Microbial infections are common clinical conditions, and antibiotics like penicillin, cephalosporin, tetracyclines, and carbapenems are effective in inhibiting microbial reproduction. However, the widespread use of antibiotics has led to the emergence of drug-resistant microbes in significant numbers. Herbal medicines offer unique natural advantages in treating microbial infections, particularly drug-resistant ones. Additionally, they have played a crucial role in the management of the COVID-19 pandemic [114]. The application of nanocrystals for antimicrobial agents is outlined in Table 6.

Berberine, an isoquinoline alkaloid found in Berberis aristata, is frequently employed in the treatment of bacterial diarrhea [115]. Due to its poor water solubility and limited permeability through intestinal epithelial cells, its oral bioavailability is around 5%. To enhance its solubility, Sahibzada et al. developed two berberine nanocrystals using the evaporation precipitation method (EPN) and antisolvent precipitation with a syringe pump method (APSP). Following nanocrystallization, berberine was in a semicrystalline state, with particle sizes of 90–110 nm for APSP and 65–75 nm for EPN. Both formulations exhibited significantly improved dissolution rates compared to crude berberine. Antimicrobial assays demonstrated that berberine nanocrystals had more potent antibacterial activity than unprocessed berberine [116].

Herpetrione (HPE), extracted from Herpetospermum caudigerum, has demonstrated potent antiviral properties. Guo et al. developed HPE nanocrystals (HPE NS) using a high-pressure homogenization method. The HPE NS exhibited a substantial increase in dissolution rate. After oral administration in rats, C_max_ and AUC_0–t_ increased by 2.45-fold and 2.49-fold, respectively, compared to crude drugs. Reduced Tmax and MRT suggested rapid intestinal absorption. Activity assessment of HPE NS indicated enhanced inhibition of HBsAg and HBeAg replication and expression, as well as more significant inhibitory effects on HBV-DNA [117].

While improving oral bioavailability is a significant advantage of nanocrystals for herbal medicines, not all drugs experience improved efficacy after conversion into nanocrystals. Thymol, known for its broad-spectrum antibacterial and antifungal activities and used in treating coughs and diarrhea [118], was examined by Zhou et al. They produced a caseinate-stabilized thymol nanocrystal through a pH-driven approach to enhance water solubility. X-ray diffraction analysis revealed that thymol nanocrystals were in an amorphous state. Unexpectedly, in antibacterial activity studies, the MICs and MBCs of thymol nanocrystals were higher than those of free thymol against four bacterial strains. This outcome contrasted with the conventional understanding and might be attributed to caseinate coating preventing thymol release, thereby reducing its contact with bacteria [119].

**Table 6:** Summary of nanocrystal formulations used in herbal medicines for antimicrobial treatment.

Extract/Compounds	Stabilizers	Preparation Methods	Particle Size	Bioavailability	Advantages	Applications	Ref.
Berberine	1% HPMC and 1% PG	Evaporative precipitation of nanocrystal (EPN) and anti-solvent precipitation with a syringe pump (APSP)	71.53 nm for EPN method, 102.62 nm for APSP method	/	BBR NPs prepared by the EPN method showed higher solubility and dissolution rate BBR NPs produced by both APSP and EPN methods showed promising activities against gram-positive and gram-negative bacteria, as well as yeasts, with NPs prepared by the EPN method showing superior results compared to those made with the APSP method.	Antimicrobial	[116]
Herpetrione	0.2% SLS and 0.3% PVP K30	High-pressure homogenization	286 ± 1.3 nm	2.45-fold in C_max_ and 2.49-fold in AUC_0–t_, and decrease in T_max_ and MRT	HPE NS increased dissolution velocity markedly; was more effective in reducing the replication and expression of HBsAg and HBeAg; and had a more significant inhibitory effect on HBV-DNA	Antiviral	[117]
Tretinoin	Bean lecithin	Antisolvent precipitation	324 nm	/	Improved drug permeation and UV irradiation stability	Acne vulgaris	[120]
Curcumin	F127 and CTAB	Wet media milling	~ 150 nm	/	Solubility and dissolution rate of CUR were significantly enhanced CUR NCs displayed low cytotoxicity against normal human kidney-2 cells CUR NCs with high positive surface charge exhibited excellent antibacterial activity compared to free CUR against E. coli and S. aureus.	Antimicrobial	[121]
Nigella sativa L. extract	1.5% PVA	Evaporative precipitation	/	/	Nanocrystals showed higher antioxidant activity than the extract Higher biofilm inhibition activity against Escherichia coli than the extract and ciprofloxacin	Antimicrobial	[122]
Thymol	Caseinate	A modified acid–base microprecipitation	79.4 nm	/	No enhancement in antimicrobial activity against four common pathogenic bacteria (Salmomella enterca, Staphylococcus aureus, Escherichia coli, and Listeria monocytogenes)	Antimicrobial	[119]

## 5. Challenges and Future Perspectives

### 5.1. Nanotoxicity of Nanocrystals

Nanotoxicity is a critical consideration in the clinical development of nanoformulations. In general, nanocrystals can potentially reduce the toxicity of drugs by increasing systemic exposure and decreasing the required dosage [123,124]. For instance, research by Gao et al. demonstrated that the LD_50_ of paclitaxel nanosuspensions stabilized with TPGs was twice that of the marketed paclitaxel for injection (98.63 mg/kg vs. 41.46 mg/kg), indicating a good tolerance of paclitaxel nanosuspensions [123]. Similarly, Zahra Karami et al. studied the oral toxicity of acetaminophen nanosuspension with a repeated dose of 100 mg/kg for 28 days, and no deaths and no abnormalities in clinical signs, body weights, or necropsy findings were observed [124].

While nanosuspensions can mitigate the inherent toxicity of drug molecules, the potential nanotoxicity of the formulation itself must also be considered. Nanoparticles offer advantages such as improved cellular uptake and targeted delivery to specific tissues, like tumors, through functionalization. For oral administration, nanocrystals tend to dissolve in the gastrointestinal fluids, and the lipophilic drug molecules are absorbed by passive diffusion. The non-absorbed stabilizers are then discharged from the body. However, functionalized nanomaterials can also interact with biological systems in unforeseen ways, potentially leading to adverse effects. For example, some stabilizers used in nanocrystal formulations, such as chitosan (open the tight junction of intestinal epithelial cells) and N-acetylcysteine (cleave the disulfide bond of mucin proteins), may interfere with intestinal mucosa function, increasing the risk of intestinal bacterial infections and inflammation. In the case of intravenous administration, non-biodegradable stabilizers can accumulate in the body, causing oxidative stress, immune reactions, and irreversible damage [125,126,127]. To mitigate these risks, biodegradable stabilizers are being explored for nanocrystal formulations.

Particle size also plays a crucial role in nanotoxicity. Particles larger than 1000 nm or micrometers can be phagocytosed by specific cells, like macrophages, in the liver, spleen, lung, and Peyer’s patches [127], resulting in limited toxic risk. In contrast, nanoparticles below 100 nm can be taken up by most cells, potentially leading to cell damage or systemic effects. Nanoparticles with sizes between 100 nm and 1000 nm are easily internalized by cells, but may be challenging to clear from the body through renal excretion. Rainer H. Müller et al. proposed a nanotoxicological classification system (NCS) to differentiate potential nanotoxicological risks that warrant careful investigation. For drug nanocrystals, via oral and external administration, the dissolution process often separates the relationship between particle size and clearance. However, for intravenous injection, when the particle size of a nanocrystal is greater than 5 μm, there is a risk of clogging capillaries.

In recent years, the cytotoxicity induced by particle shape has gained attention. For instance, needle-shaped particles were found to induce transient disruption of cell membranes, although cell recovery was identified after 48 h [128]. Shape effects on physiological responses were studied by Zhang et al. using PLGA-PEG nanoparticles, revealing that needle-shaped nanoparticles caused DNA fragmentation and cell death by disrupting lysosomes and activating apoptosis pathways, which was not observed with spherical nanoparticles [129]. Shape heterogeneity is a feature of nanocrystals due to their unique crystal lattices. Drug nanocrystals can be developed into various shapes, including spherical, rod-like, flaky, and oblong [11], which may impact their toxicological profiles. Additionally, different shapes may influence the maximum tolerated doses, affecting long-term toxicity. It has been reported that PEGylated hydroxycamptothecin nanorods have a higher lethal dose than PEGylated hydroxycamptothecin nanoparticles [64]. While research on the nanotoxicity caused by different shapes of drug nanocrystals is limited, it is an aspect that should not be ignored during the development of herbal medicine nanocrystals.

### 5.2. In Vivo Fate of Herbal Medicine Nanocrystals

Understanding the in vivo fate of herbal medicine nanocrystals is crucial for their successful application. While the primary purpose of nanocrystal technology is to enhance drug dissolution and bioavailability, research has shown that nanocrystals can be absorbed intact through intestinal epithelial cells [11] and lymphatic transport [14], as well as through systemic circulation after intravenous administration. To track nanocrystals in vivo, innovative approaches, such as fluorescent labeled hybrid nanocrystals, have been developed. For example, ACQ probes have water-quenching sensitivity, which means that the fluorescent signal can be detected when the nanocrystals are intact, and the signal disappears when the nanocrystals are dissolved [130]. The use of aggregation-caused quenching (ACQ) fluorescent probes embedded within drug nanocrystals allows for real-time monitoring of their dissolution and fates. Studies with CUR hybrid nanocrystals have found that they are mainly captured by the mononuclear phagocytic system, accumulating in the liver and lungs, with a portion persisting in the blood for up to 48 h before accumulating in reticuloendothelial organs and tissues. The prolonged circulation may result from the surface coating of F68. Surface modification, particle size, and particle shape have all been identified as factors that can alter the in vivo fate of nanocrystals. In the case of quercetin hybrid nanocrystals (QT-HNCs) via the oral route [131], QT-HNCs can be retained in the gastrointestinal tract for 12–16 h, and then distribute in the liver, spleen, lung, and kidney with particle size-related discriminating biodistribution. At 12 h, the biodistribution of QT-HNCs in different tissues reached its maximum. In our previous study, we proposed that particle shape was also a non-ignored factor that can alter the in vivo fate of nanocrystals [12]. We found that rod-shaped nanocrystals had longer retention times in the gastrointestinal tract. To sum up, surface modification, particle size, and particle shape should be fully considered in order to optimize the biodistribution and therapeutic effects of herbal medicine nanocrystals.

### 5.3. Potential Advantages of Overcoming Drug Resistance

Drug resistance is a significant challenge in cancer and antimicrobial treatments. Compared with chemotherapeutic drugs, herbal medicines offer advantages in terms of reversing multidrug resistance (MDR) in tumors, regulating immunity, and reducing non-target toxicity. Qiao et al. developed a hybrid nanocrystal containing hydroxycamptothecin (HCPT, an antitumor natural ingredient) and quercetin (QUR, a natural flavonoid compound with anti-MDR and immunomodulatory effects) [132]. The hybrid nanocrystals (HQ-NPs) were quasi-spherical in morphology, which was different from the HCPT nanosuspension (spherical-like) and QUR suspension (rodlike). The molecular dynamics study showed that the integration of HCPT and QUR was attributed to the strong π–π stacking and hydrophobic interactions of the planar structures. In H22 tumor-bearing mice, HQ-NPs showed similar accumulation of HCPT in tumor sites and antitumor effects compared with the HCPT nanosuspension. However, the concentration of HCPT in HQ-NPs was only 3/5 of that in the HCPT nanosuspension, indicating that HQ-NPs had enormous potential to overcome MDR. This result may be because QUR accelerated the cellular uptake of HQ-NPs by inhibiting P-gp, although the antitumor effect of QUR was weak.

Antibiotic resistance is another urgent challenge to be solved worldwide. The emergence of antibiotic resistance is mainly due to the widespread abuse of antibiotics. It has been reported that the most promising herbal medicines to be used alone against multidrug-resistant bacteria are *Piper betle*, *Glycyrrhiza glabra*, and *berberine* [133]. Nanocrystals have exhibited increased antibacterial effects for these drugs compared to unprocessed ones, as described in Section 4.5. In other situations, herbal medicines in combination with antibiotics have expanded the spectrum against multidrug-resistant bacteria [133]. The underlying mechanisms may be that herbal medicines could inhibit the efflux pump; modify the bacterial cell wall and/or membrane; inhibit the cell division protein-filamenting, temperature-sensitive Z-ring; and inhibit protein synthesis and gene expression. Harsha Kathpalia et al. investigated the effect of a combination of atovaquone nanocrystals–proguanil–artesunate on antimalarial therapy. Atovaquone nanocrystals were proven to be effective alone at 1/80th the therapeutic dose. In the triple combination, a complete cure was observed with atovaquone nanosuspension in combination with proguanil at 1/80th the therapeutic dose of each and 1/5th the therapeutic dose of artesunate, indicating that nanocrystals can reduce the therapeutic dose and delay the occurrence of antibiotic resistance [134]. Even though the therapeutic dose was dramatically reduced when applying nanocrystal technology, the optimal dosing ratio for herbal medicines and antibiotics needs to be further determined.

### 5.4. Herbal Ingredients as Stabilizers of Nanocrystals

Due to the potential toxicity of synthetic polymers or surfactant-based stabilizers, natural sources of stabilizers have attracted increasing attention. Saponins extracted from natural plants are widely used in food and beverages as foaming agents and emulsifiers [135] due to their amphipathic structure, with hydrophobic triterpene groups and hydrophilic sugar chains. Glycyrrhizin is a natural triterpenoid saponin. Chen et al. used glycyrrhizin to stabilize andrographolide nanocrystals (AGE-NS). Under the interfacial properties and electrostatic effects of glycyrrhizin, AGE-NS could be entrapped in its network structure [136]. Li’s group prepared hesperidin nanocrystals using tea saponins as a novel multifunctional stabilizer [137]. Compared with synthetic stabilizers, tea saponins have stabilization efficiency at low concentrations. In addition, they could prevent the aggregation of nanocrystals in the freeze-drying process by acting as a cryoprotectant. In another study, the researchers used gypenosides, a tetracyclic triterpenoid compound extracted from *Gynostemma pentaphyllum*, to stabilize quercetin nanocrystals (QUE-NS) [138]. Compared with glycyrrhizin, tea saponins, and other synthetic stabilizers, QUE-NS stabilized with gypenosides showed the smallest particle size and a narrow size distribution (PDI < 0.1). Lentinan is a natural β–glucan with various bioactivities, such as antitumor effects. Suo et al. proved that lentinan could stabilize regorafenib nanocrystals by forming hydrogen bonds and hydrophobic interactions between the two molecules. They also found that lentinan could enhance the in vitro anticancer activity and oral bioavailability of regorafenib [139]. With the discovery of an increasing number of natural stabilizers, the dual roles of herbal active ingredients have been explored. However, whether natural stabilizers are superior to synthetic stabilizers in terms of therapeutic efficacy is not yet fully understood. Meanwhile, the toxicity of natural stabilizers needs further investigation.

## 6. Conclusions

In conclusion, herbal medicine’s appeal in disease therapy is rising due to its diverse pharmacological effects and multi-target capabilities. However, the poor water solubility and potential toxicity of many active herbal ingredients have hindered their clinical application. Nanocrystals offer a promising solution for delivering insoluble drugs, offering benefits such as high drug loading, improved solubility, enhanced bioavailability, and reduced systemic toxicity. The successful commercialization of products like Emend^®^, Tricor^®^, Rapamune^®^, and Celevrex^®^ underscores the potential and conversion opportunities of nanocrystals. However, the application of nanocrystals in herbal medicine is still in its early stages.

Currently, the evaluation of herbal medicine nanocrystals often focuses on physical characterization, in vitro dissolution, and bioavailability. However, aspects such as physical and chemical stability, absorption, and therapeutic mechanisms deserve more attention. Integrating the clinical theories of herbal medicine with nanocrystal technology is essential for its comprehensive development. Moreover, research on the in vivo fate of herbal medicine nanocrystals has primarily been concentrated on oral and intravenous administration, with limited exploration in pulmonary, ophthalmic, and intranasal delivery methods. Nanotoxicity is an important consideration in the development of herbal medicine nanocrystals, necessitating thorough investigation to ensure their safety. To avoid the toxicity caused by synthetic stabilizers, naturally derived stabilizers may be a novel development direction for the next generation of nanocrystals.

In conclusion, the combination of herbal medicines with nanocrystals holds promise for overcoming drug resistance and improving therapeutic outcomes. However, thorough investigations are needed in order to understand the interactions between nanocrystals, herbal medicines, and biological systems to ensure safe and effective treatment.

## Figures and Tables

**Table 1:** Advantages and limitations of the preparation method of **herbal medicine** nanocrystals.

Preparation Method	Advantages	Limitations	Subdivide Method
Bottom-up	simple operation most cost-effective more suitable for intravenous administration of anticancer compounds	residual organic solvent may cause toxicity not suitable for drugs which are difficult to dissolve in organic solvents the nucleation and crystal growth processes are difficult to control	anti-solvent precipitation supercritical fluid technology evaporative precipitation into aqueous solution
Top-down	no need to use an organic solvent easy to scale up production	high cost residues of milling media	wet media milling high-pressure homogenization
Combinative methods	smaller particle sizes relatively short times	complicated process residual organic solvent is hard to remove	NanoEdge ™ H69 technology H42 technology H96 technology

**Table 4:** Summary of nanocrystal formulations used in herbal medicines for cardiovascular diseases.

Extract/Compounds	Stabilizers	Preparation Methods	Particle Size	Bioavailability	Advantages	Applications	Ref.
Berberine	TPGS	High-pressure homogenization	73.1 ± 3.7 nm	/	Exhibited superior hypoglycemic, total cholesterol (TC), and body weight reduction effects compared to bulk Ber and metformin (Met, 300 mg/kg).	Antidiabetic effect	[98]
Ginkgo Lactone/ginkgolide	5% P 188 and 5% HPMC	High-pressure homogenization	254 ± 2.8 nm	Twofold higher in C_max_ and AUC_0–t_ for three ginkgolides	Exhibited a significantly higher antiplatelet aggregation effect	Antiplatelet aggregation	[99]
Honokiol	Bovine serum albumin (BSA) and PVP	Antisolvent sonoprecipitation	116.2 ± 2 nm	Honokiol nanocrystals improved the oral bioavailability in rats by 3.94-fold in C_max_ and 2.2-fold in AUC_0–t_	Honokiol was released more quickly in vitro from nanocrystals, with no burst release. Honokiol nanocrystals improved the oral bioavailability. After intraperitoneal administration, Honokiol nanocrystals could dramatically alter the biodistribution, resulting in much higher drug levels and tissue bioavailability in the blood, heart, and brain.	Cardio-cerebro-vascular system	[97]
Betulin	0.5% Tween 80	Antisolvent precipitation	~110 nm	The oral bioavailability of the betulin nanocrystal was 2.21 times that of raw betulin.	Higher dissolution rate, solubility, and bioavailability of botulin nanocrystal compared with raw botulin. Excellent hypoglycemic effect compared with raw betulin	Diabetes mellitus	[100]
Ursolic acid	2% of PVA	Nanoprecipitation	246.4 ± 4.21 nm	/	Ursolic acid nanocrystal showed a significant reduction in elevated blood glucose level in a dose-dependent manner with prominent lipid-lowering and antioxidant effects.	Type II diabetes	[101]
Breviscapine	Soybean phospholipid	Antisolvent sonoprecipitation	303.7 ± 7.3 nm	/	Breviscapine nanocrystal displayed good stability, increased solubility, and better in vitro release	Cardiovascular and cerebrovascular diseases	[102]

## Data Availability

Not applicable.

## Abbreviations

10-HCPT	10-hydroxycamptothecin
ADG	Andrographolide
ANDA	Abbreviated New Drug Application
ART	Artemisinin
BCS	Biopharmaceutics classification system
BSA	Bovine serum albumin
CUR	Curcumin
DSPE	1,2-Dioleoyl-sn-glycero-3-phosphoethanolamine
EPAS	Evaporative precipitation into aqueous solution
GNA	Gambogenic acid
HPC	Hydroxypropyl cellulose
HPH	High-pressure homogenization
HPMC	Hydroxypropyl Methyl Cellulose
ICT	Icaritin
IND	Investigational New Drug
MDA	Malondialdehyde
NDA	New Drug Application
NRG	Naringenin
P188	Poloxamer 188
PG	Propylene glycol
PVA	Polyvinyl acetate
PVP	Polyvinylpyrrolidone
PVP/VA	Polyvinylpyrrolidone/vinyl acetate
QT	Quercetin
SCF	Supercritical fluid technology
SDS	Sodium dodecyl sulfate
SOD	Superoxide dismutase
TPGS	D-α-Tocopherol polyethylene glycol succinate
WMM	Wet media milling
